# Genotoxicity of *Paragonimus heterotremus* Infection in a Rat Model of Simultaneous Pulmonary and Hepatic Paragonimiasis

**DOI:** 10.3390/biomedicines9091180

**Published:** 2021-09-08

**Authors:** Galina N. Chelomina, Sergey P. Kukla, Viktor P. Chelomin, Pham N. Doanh

**Affiliations:** 1 Federal Scientific Center of the East Asia Terrestrial Biodiversity, Far-Eastern Branch of Russian Academy of Science, Vladivostok 690022, Primorsky Krai, Russia; 2 V. I. Il’ichev Pacific Oceanological Institute, Far East Branch of the Russian Academy of Sciences, Vladivostok 690022, Primorsky Krai, Russia; chelomin@poi.dvo.ru; 3 Institute of Ecology and Biological Resources, Graduate University of Sciences and Technology, Vietnam Academy of Science and Technology, Hanoi 100000, Vietnam; pndoanh@yahoo.com

**Keywords:** paragonimiasis, oxidative stress, genotoxicity, DNA damage, comet assay

## Abstract

Parasites cause numerous health issues in humans, eventually leading to significant social and economic damage; however, the mechanisms of parasite-mediated pathogenesis are not well understood. Nevertheless, it is clearly evidenced that cancerogenic fluke-induced chronic inflammations and cancer are closely associated with oxidative stress. (1) Methods: The *Paragonimus heterotremus* infection’s genotoxic potential was assessed in a rat model of simultaneous pulmonary and hepatic paragonimiasis by the alkaline version of single-cell gel electrophoresis (comet assay). Statistical analysis of comet parameters was based on the non-parametric Mann–Whitney U test. (2) Results: A clear and statistically significant increase in DNA damage was detected in the helminth-exposed group versus the control rats and the tissue areas adjacent to the parasite capsule versus remote ones; however, differences in DNA damage patterns between different tissues were not statistically significant. Infection resulted in up to 40% cells with DNA damage and an increased genetic damage index. (3) Conclusions: The data obtained contribute to understanding the pathogenesis mechanisms of paragonimiasis, suggesting oxidative stress as the most likely reason for DNA breaks; these findings allow us to consider *P. heterotremus* as a potentially cancerogenic species, and they are important for the monitoring and treatment of paragonimiasis.

## 1. Introduction

As a result of their global distribution, parasites cause numerous health issues in humans and animals, eventually leading to significant social and economic damage in all countries. About 15–18% of malignancies worldwide are attributed to viral, bacterial, and parasitic infections, including helminth infection (e.g., *Schistosoma haematobium* is the causative agent of urinary bladder cancer; *Clonorchis sinensis* and *Opisthorchis viverrini* are unarguable risk factors for cholangiocarcinoma [1,2,3]. However, on the whole, the role of parasitic infection in the development of human cancer diseases remains underestimated, and its dual impact (an induction and suppression) on tumorigenesis is possible [4,5,6,7].

Paragonimiasis is a zoonotic disease caused by lung flukes of the genus *Paragonimus.* Almost 23 million people worldwide are infected by these species, which creates a global burden of 1,048,937 disability-adjusted life years (DALYs), with 292 million people being at risk. On the WHO list of the 24 most prevalent and important food-borne parasites, the *Paragonimus* species is ranked 14th [8]. *Paragonimus heterotremus* is the most significant species as a causative agent of paragonimiasis in Vietnam, Thailand, Lao PDR, and some regions of China [9,10]. Paragonimiasis is usually characterized by inflammatory lung injury, with its clinical manifestations depending on the stage of infection; in the chronic stage, its symptoms resemble tuberculosis and lung cancer [9]. Although *Paragonimus* flukes primarily affect the lungs, they can form significant lesions and worm-containing granulomas in other body regions (e.g., liver, spleen, brain, skin, etc.). Ectopic paragonimiasis is most frequently observed (as a rule, simultaneously with the lung form) in cases of *P. skrjabini*, *P. mexicanus*, and *P. heterotremus* infection [9]. The mechanisms of the parasite-mediated pathogenesis in paragonimiasis are less well understood compared with those of cancerogenic flukes. Therefore, novel knowledge about host pathology and underlying mechanisms is crucially needed to provide effective treatments for parasitic infections and, consequently, better human health.

During invasion, helminths constantly secrete and excrete metabolic products (ESPs, excretory-secretory products) into the intercellular space, which plays a key role in parasite–host interactions. ESPs can be highly immunogenic and toxic themselves as well as through their interactions with other molecules. ESPs promote proliferation and suppress apoptosis of malignant or abnormal cells, and they induce genome-wide DNA methylation changes, genome instability, and alterations in transcriptomic, proteomic, and miRNA profiles through various proteins [3,11,12]. 

In terms of cancerogenic flukes, it is clearly evidenced that parasite-induced chronic inflammations and cancer are closely associated to reactive oxygen species (ROS) and reactive nitrogen species (RNS), with endogenous DNA damage being the major etiological factor in human cancer. At a low level, free radicals act as secondary messengers involved in the signaling, differentiation, proliferation, apoptosis, and modulation of transcription factors. The overproduction of ROS and RNS, targeted primarily against invading pathogens, contributes to heavy cellular injury and several disorders in humans. Excess production of intracellular free radicals can lead to oxidative modification of macromolecules (DNA, proteins, and lipids) and DNA alterations (e.g., strand breaks, base-free sites, deletion, frameshift, etc.) in a host’s organism and is associated with genome instability. There are also indirect effects of ROS/RNS exerted by inhibiting the DNA repair enzymes and/or metabolic activation of known carcinogens [3,13,14]. Thus, in humans and animals infected by parasites, DNA molecules and DNA repair systems are targets for both the pathogen’s ESPs and the parasite-induced ROS/RNS of the host, both of which lead to DNA breaks.

The DNA comet assay (single-cell gel electrophoresis) is a rapid and effective method for detecting DNA damage, such as single- and double-strand breaks and incomplete repair sites, at the single-cell level. This method is widely used in various fields of biological, ecological, and medical research [15,16,17]. As a reliable tool for the study of qualitative and quantitative DNA damage in vivo and in vitro, we first used a single-cell comet assay to measure the genotoxic potential of *P. heterotremus* infection in a rat model of simultaneous pulmonary and hepatic paragonimiasis. Along with other parameters calculated by this method, DNA content in the tail (%DNAt), as the best indicator of induced DNA damage, was applied to quantify DNA strand breaks and assess the degree of damage in infected and uninfected animals. The study aimed to elucidate whether *P. heterotremus* infection of rats induces DNA damage (due to oxidative stress), resulting in DNA breaks in the organs of parasite localization, as well as how much the intended damaging effect varies between different tissues and at different distances from the parasite capsule. 

## 2. Materials and Methods

### 2.1. Rat Infecting

*Paragonimus heterotremus* metacercariae from freshwater crabs *Potamiscus tannanti* (Rathbun, 1904), collected in the north of Vietnam, were fed to *Rattus norvegicus* (Wistar albino rats, laboratory line); rats were dissected at 17 days (when larvae migrated) and 2 months after infection (when larvae were encysted in lung and liver and juvenile flukes began egg secreting) as described [18]. All procedures in the present work were performed in accordance with the international regulations and guidelines of experimental animal studies (Directive 2010/63/EU of the European Parliament and of the Council of 22 September 2010) and were approved by the Commission on Bioethics at the Federal Scientific Center of the East Asia Terrestrial Biodiversity, Far-Eastern Branch of Russian Academy of Science (protocol code 2020sm06 and date of approval 3 March 2020), Vladivostok, Russia.

### 2.2. Preparation of Single-Cell Suspensions

The liver and lung tissue samples (Figure S1) were removed from the organ sections adjacent to and remote from the parasite capsule, and washed four times in 5 mL of ice-cold calcium-magnesium-free saline (CMFS) consisting of 0.050 M Tris-HCl (Sigma-Aldrich, St. Louis, MO, USA), pH 7.4, 0.14 M NaCl (ITW Reagents, Barcelona, Spain), and 5 mM EDTA (Sigma-Aldrich). These samples were then macerated with scissors for 5 min in a volume of 2 mL CMFS, and the individual cell suspensions were transferred to a flask, and 10 mL CMFS was added. The suspension was agitated gently for 1 h on ice in the dark, and the large clusters of cells were removed by filtering the suspension through a 40 μm sieve. The isolated cells were centrifuged at 1500 rpm (250× *g*) for 5 min, the supernatant was removed, and the cells were resuspended in the remaining CMFS to a concentration of 10^5^ cells/mL. The cell suspensions primarily contained hepatocytes and pneumocytes; the method used does not provide for the disintegration of connective tissue cells [19].

### 2.3. Alkaline Single-Cell Gel Electrophoresis (Comet) Assay

Single-cell gel electrophoresis (comet) was applied to the liver and lung cell suspensions to determine the level of DNA strand breakage based on the procedure described [19]. Alkaline unwinding was performed for 15 min in the electrophoresis solution (0.3 M NaOH (ITW Reagents) and 1 mM EDTA (Sigma-Aldrich), followed by 15 min of electrophoresis at 300 mA and 2 V/cm. The slides were stained with ethidium bromide (20 μg/mL) (Sigma-Aldrich) and examined with a scanning fluorescence microscope AxioImager A1 (Carl Zeiss AG, Oberkochen, Germany), equipped with an AxioCam MRc digital camera (Carl Zeiss AG, Oberkochen, Germany). On each slide, about 100 randomly chosen cell nuclei were examined automatically with V 1.2.2. CASP program (CASPlab, Wroclaw, Poland), which allowed for the calculation of various parameters of the comets, thus indicating the level of DNA damage.

A total of 100 nuclei from each of the two replicates were examined and classified in one of the five damage classes, according to the migration distance and the fluorescence rate between the head and the tail of the nucleus: Class 0, intact nucleus with no migrated fragments (<5% fragmented DNA); Class 1, dense nucleus, with slight DNA migration forming a small tail (5%–20% fragmented DNA); Class 2, tail extending from the nucleus, with a weaker fluorescence than Class 1 (20%−40% fragmented DNA); Class 3, comets with a clear tail that may reach full length (40%–75% fragmented DNA); and Class 4, nucleus, when present, is small and completely separated from the tail (>75% fragmented DNA). In addition, the classes were subdivided into smaller groups (with an interval of 5% DNAt) to gather a more detailed understanding of the changes occurring in cells and to identify the proposed differences.

The level of DNA damage was evaluated as follows: (1) the percentage of DNA in the comet tail (% DNAt), (2) the tail length (Lt), and (3) an index of DNA integrity or the genetic damage index (GDI), calculated for each of the experimental and control sampling sets based on the number of comets from each class: [C1 + (2 × C2) + (3 × C3) + (4 × C4)]/(C0 + C1 + C2 + C3 + C4) [20].

### 2.4. Statistical Analysis

Statistical analysis was carried out using the STATISTICA software V. 7(TIBCO Software Inc., Palo Alto, CA, USA). First, data were tested for normality (Shapiro–Wilk test) and homogeneity of variance (Leven test), with visual examination of QQ-plots. Due to the invalidation of at least one of the tests, the non-parametric Mann–Whitney U test was employed for comparison of different groups. A significance level of α = 0.05 was set for all statistical analysis.

## 3. Results

**Figure 1:**
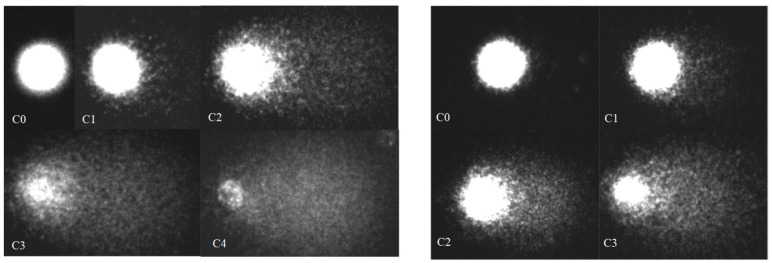
Comet images of the lung (**left**) and liver (**right**) cells in control and *Paragonimus heterotremus*-infected rats for different DNA damage classes (0–4).

**Figure 2:**
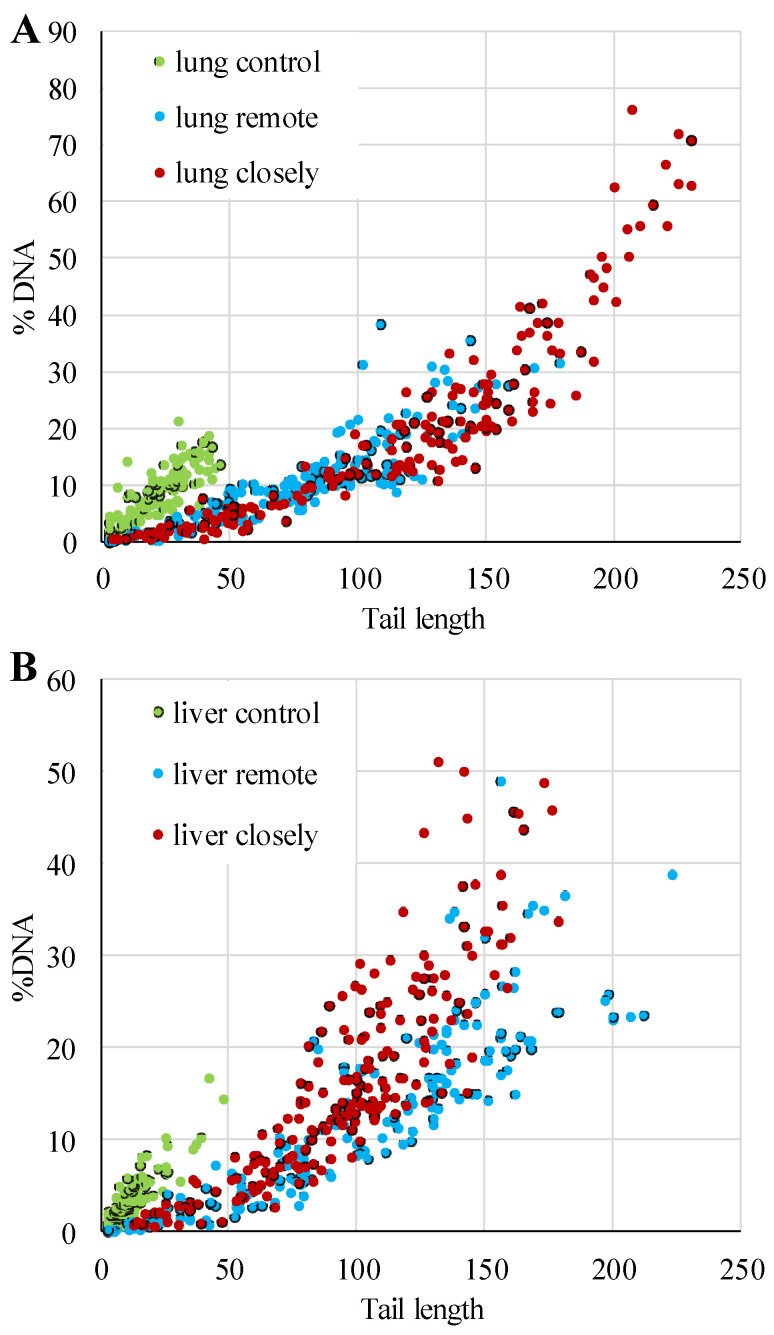
Correlation between the fraction of migrating DNA and tail length in comets for the lung (**A**) and liver (**B**) cells of control and *Paragonimus heterotremus*-infected rats. The percentage of DNA in the tail of the comet and the tail length was measured on 100 nuclei per sample in two replicates.

In single-cell electrophoresis, intact DNA molecules formed a symmetrical bright nucleus with the surrounding “halo,” represented by loops of high-polymer DNA released into agarose. DNA molecules with a strand break formed a comet-like image due to genome degradation and migration of low-polymer DNA fragments (Figure 1). The comet parameters %DNAt and Lt, reflecting the degree of DNA damage to the pulmonary and hepatic cells, were widely ranged in the experimental group of rats infected by *P. heterotremus* and had a narrow range of values in the control groups. Furthermore, the ratio of these indices differs between the control and experimental animals (linear versus exponential), suggesting alterations in the host’s physiological status under infection (Figure 2).

**Figure 3:**
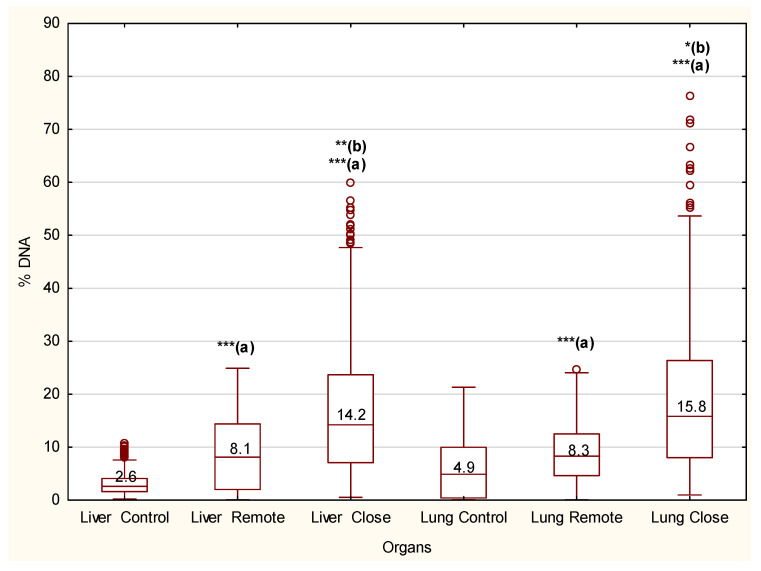
The percentage of DNA in the tail of the comet for the lung and liver cells of control and *Paragonimus heterotremus*-infected rats. Boxplots represent the median (line), 25–75% percentiles (box), and min-max (whiskers). Asterisks (***, **, *) are indicative of significant differences from the corresponding control (a) or differences between remote and close to parasite capsule cells (b) (*p* = 0.000, *p* = 0.0003, *p* = 0.005). Differences between replicates are statistically nonsignificant (*p* = 1.0). Statistical differences were based on the Mann–Whitney U test. o represents outlier.

The genotoxic effects due to parasite exposure are strongly statistically supported (Figure 3). Relative to control, there were significantly induced alterations in the lung and liver DNA, close and remote from the parasite capsule (*p* = 0.000). The levels of DNA damage were also statistically significant in comparisons of close and remote cells both in the lung and liver (*p* = 0.00029 and *p* = 0.00450, respectively). However, no statistically significant differences in DNA damage were observed between tissues; for comparisons of control, remote, and close cells, *p* = 0.155, 0.571, and 0.211, respectively. Median values indicate approximately 2–3 times increase of DNA percentage in the tail for remote cells and 3–5 times increase for close cells.

**Figure 4:**
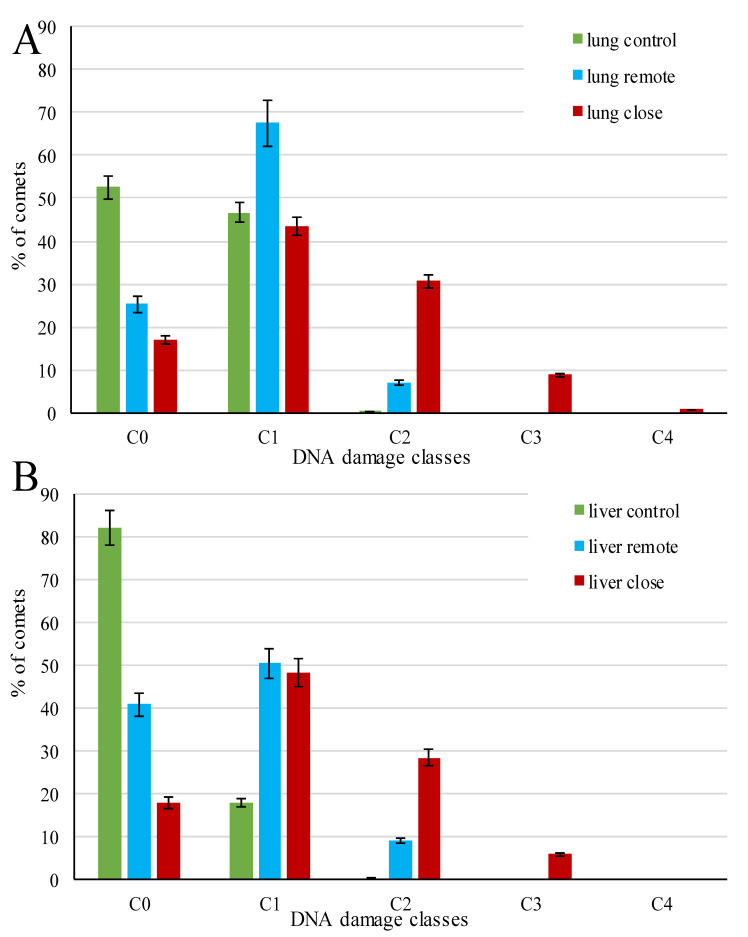
Cell gradation by DNA damage classes in the lung (**A**) and liver (**B**) tissues of control and *Paragonimus heterotremus*-infected rats. Histograms represent mean value (boxes) and min-max (whiskers). Differences between replicates are statistically nonsignificant (*p* = 1.0).

The distribution of cells over the standard classes of DNA damage also demonstrated the heterogeneous response among cells of the same tissues, in different tissues, and at different distances from the infection source within the same tissue type (Figure 4). The control group cells were intact or had low-grade damage, referring to Classes C0 and C1, respectively. They were characterized by zero or minimal DNA migration in gel electrophoresis, though the ratio between these classes varied due to the higher content of C1 in the lung control. In areas of lung and liver tissue remote from the parasite capsule, the major parts of cells (about 90%) were represented by Classes C0 and C1; however, in contrast to control, most of the comets were represented by Class C1, and the remaining cells (7–9%) exhibited medium DNA damage (Class C2). In the lung and liver tissue areas adjacent to the parasite capsule, significantly increased DNA damage was observed. In both tissues’ cells, the proportion of Classes C0+C1 decreased by about a third, while that of C2 increased 3–4 times compared to the remote tissue areas. For the rest of the comets, approximately 6% hepatic and 9% pulmonary cells were represented by Class C3, i.e., characterized by a high DNA damage level. However, Class C4, combining cells with the greatest damage, was represented only by single lung cells.

A more detailed analysis of cells’ distribution by the degree of their damage (Supplementary Figure S2) revealed that comets with 30–40% DNA content in the tail (C2) were formed only in the cells adjacent to the parasite capsule in both tissues (i); comets with 60–80% DNA in the tail (C3 and C4) formed only among lung cells adjacent to parasite capsules (ii); intact cells (C0) with DNA content in the tail up to 1% formed only in liver cells (iii).

DNA damage caused by parasite infection in different tissue cells showed a statistically significant (*p* < 0.05) increase in GDI (1.7–6.8 times) when compared to the control group of rats unexposed to the parasite (Table 1). The GDI level was mostly aligned with Classes 0 and 1 in the uninfected rats and Classes 1–3 in infected rats.

## 4. Discussion

At any given time, a certain part of the genome is damaged; only metabolism-generated ROS/RNS can cause approximately 10,000 DNA lesions per cell per day in humans and 100,000 lesions per cell per day in rats. However, in the physiological state, the generation of DNA breaks is balanced by subsequent DNA repair [21,22]. In the beginning stage of paragonimiasis, changes are found for the physiological norm in Classes C0 and C1. Since Class C1 includes DNA regions with incomplete repair and transcriptionally active regions, its increase can indicate the earliest host response to helminth infection that is associated with the activation of certain genes and a slight imbalance between DNA damages and their repair.

Cells with medium DNA damage (Class C2) are typical under stress due to various changes in environmental conditions. Thus, C2, as the main class of DNA damage, and its recovery by reoxygenation, was found in gill cells of scallops exposed to anoxic stress [19]. Therefore, C2 (which is also the main class in our experiment) can be considered an indicator of cellular stress caused by parasitic infection when damaged tissues have a high chance of recovery. However, even the normal repair of DNA breaks can occasionally cause heritable silencing of CpG-island-containing promoters; with the contributions of stress-related proteins, breaks can lead to the aberrant CpG island methylation that is frequently associated with gene silencing in cancer. Errors in the repair (with its pathways determined by cell cycle phase and cell type) of double DNA strand breaks can cause mutations and chromosome instability, leading to cancer or cell death [23].

The emergence of Class C3 usually indicates high DNA damage, suggesting a damage rate greater than the rate of recovery observed during oxidative stress. Class C4 has an extremely high DNA damage level when DNA is almost entirely in the comet tail, and almost no cell recovery is possible, with cell death being the most likely event. Along with the lighter injuries described above, such damages were recorded for different animals; for example, after exposure to X-rays, benzene, heavy metals, or other toxins [24,25], and also after exposure to cancerogenic parasites such as *Toxoplasma gondii*, *Helicobacter pylori* [26,27], and *Taenia solium*, as well as officially non-carcinogenic *Hymenolepis nana*, *Toxocara canis*, and *Trichinella spiralis* [28,29].

DNA damage is known to be time dependent, i.e., it can accumulate over time. For example, such an effect was reported for infections caused by *Taenia solium* in a hamster model of taeniasis [28], by *Toxoplasma gondii* in experimental toxoplasmosis in mice [26], and by *Opisthorchis felineus* in a hamster model of opisthorchiasis [7]. DNA damage also depends on the concentration of parasitic proteins; such damage has been observed in vitro for a co-culture of donor blood lymphocytes and protein somatic products from helminths [29]. These facts can explain why C4 and C3 are only found in cells adjacent to a parasite capsule. Given the influence of a parasite on a host organism as a whole, the accumulation of DNA damage can aggravate the severity of concomitant diseases and contribute to the emergence of new ones, including malignant transformation. The accumulation of DNA damage can occur either due to an increase in the number of events damaging DNA or due to a decrease in DNA repair, which is an issue that needs to be resolved in the future.

Another issue that needs to be addressed is whether the accumulation of oxidative damage or cellular apoptosis and/or necrosis predominates in chronic paragonimiasis; moreover, among *P. heterotremus* ESPs, are there specific molecules that prevent tumorigenesis? Indeed, despite the infection genotoxicity (recorded in this paper) in rats with chronic paragonimiasis, numerous histopathological changes in the lung and liver tissues were observed (including necrosis), with no malignant transformations [18]. These facts refer us to discussed data on the dual role of parasitic infections and antitumor effects of some molecules produced by helminths and their use as potentially effective candidates for drugs against cancer [5,6].

In general, the obtained results on DNA damage are comparable to those of clinical observations in patients with diseases of high prevalence, including chronic obstructive pulmonary disease and breast cancer. They have, on average, 2–3 times higher levels of DNA strand breaks in leukocytes versus healthy controls [24]. The genotoxicity of *P. heterotremus* infection is similar to that reported for fascioliasis. At the acute stage of the disease in rabbits, the average comet tail length was significantly greater (several times) in liver cells of the animals infected by *F. gigantica* versus controls. This liver fluke was proposed to be considered a potentially cancerogenic species [2]. Changes in comet parameters were also observed in other parasitological infections, e.g., toxoplasmosis [26] and *Helicobacter pylori* infections [27].

## 5. Conclusions

This study has shown that *P. heterotremus* infection possesses genotoxic potential in a small mammalian model, and this potential is most likely due to oxidative stress and an inflammatory environment. Therefore, this fluke can be considered a potentially cancerogenic species that matches existing opinions regarding the underestimated and dual role of parasitic infections in the development of human cancer diseases. Further studies regarding pathology in paragonimiasis and its underlying molecular mechanisms are required to treat parasite-associated diseases more successfully. The exploration of the genotoxic effect of this fluke in the dependence on tissues and cell types, as well as the intensity and duration of infection, could be of particular interest.

## Figures and Tables

**Table 1:** Genetic damage index (GDI) indices of the lung and liver cells in the control and *Paragonimus heterotremus*-infected rats.

Index	Control		Lung		Liver	
	Lung	Liver	Close	Remote	Close	Remote
GDI	0.48 ± 0.034	0.18 ± 0.013	1.33 ± 0.093	0.82 ± 0.057	1.23 ± 0.0161	0.68 ± 0.048

## Data Availability

The datasets used during the current study are available from the corresponding author on reasonable request.

## Supplementary Materials

The following are available online at https://www.mdpi.com/article/10.3390/biomedicines9091180/s1.
