# Supplementary figures and images for: Genotoxicity of Paragonimus heterotremus Infection in a Rat Model of Simultaneous Pulmonary and Hepatic Paragonimiasis
Source: Biomedicines. 2021 Sep 8;9(9):1180. doi: 10.3390/biomedicines9091180 (PMC8469942; doi:10.3390/biomedicines9091180)

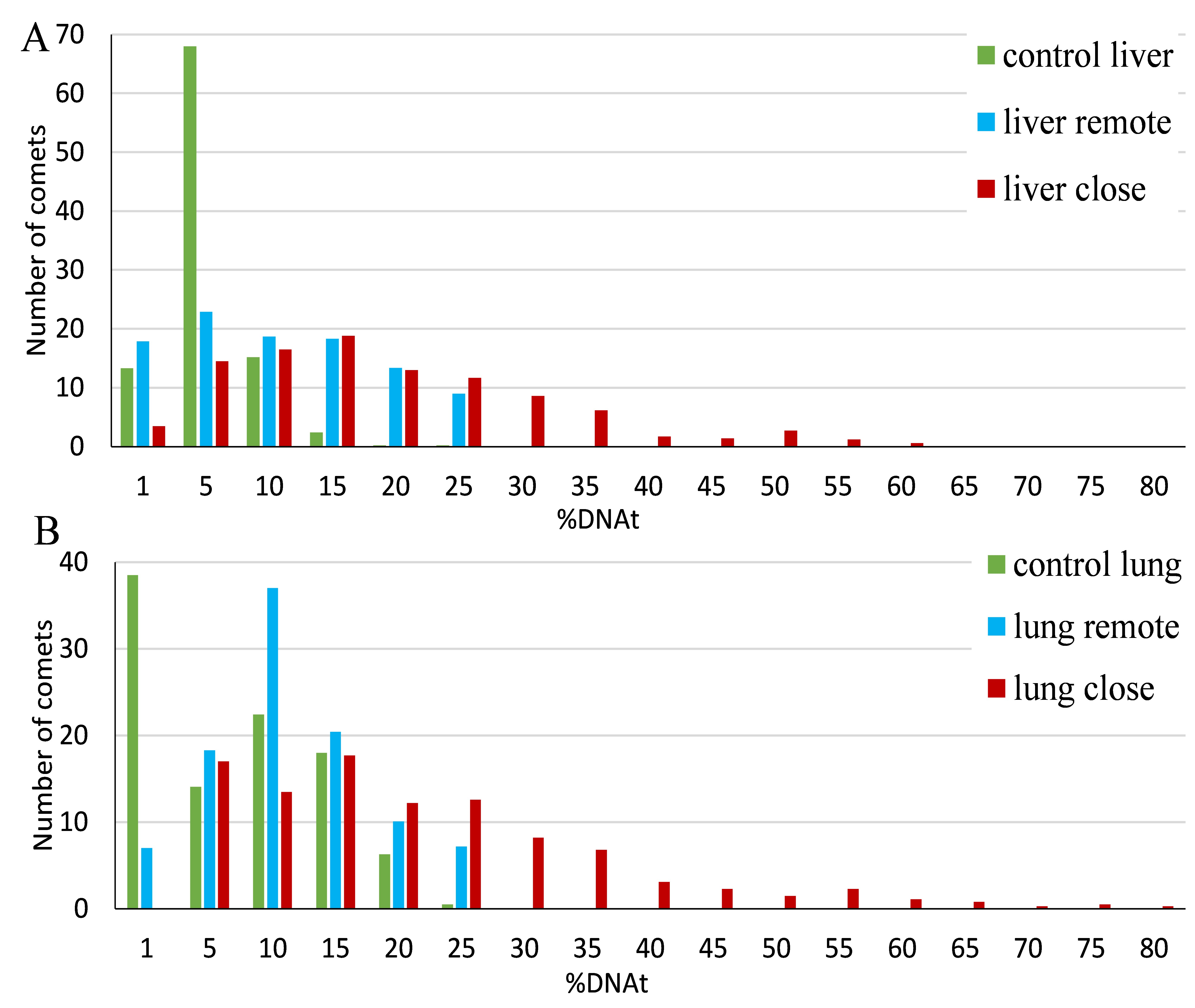

Supplement: Supplementary file 1 [file biomedicines-09-01180-s001.zip › Figure S2.tif]
